# CD147 overexpression on synoviocytes in rheumatoid arthritis enhances matrix metalloproteinase production and invasiveness of synoviocytes

**DOI:** 10.1186/ar1899

**Published:** 2006-02-08

**Authors:** Ping Zhu, Ning Lu, Zhan-guo Shi, Jun Zhou, Zhen-biao Wu, Yong Yang, Jin Ding, Zhi-nan Chen

**Affiliations:** 1 Department of Clinical Immunology, Xijing Hospital, Fourth Military Medical University, Xi'an, 710032. Shaanxi Province, PR China; 2 Cell Engineering Research Center & Department of Cell Biology, Fourth Military Medical University, Xi'an, 710032, Shaanxi Province, PR China

## Abstract

Macrophage-like synoviocytes and fibroblast-like synoviocytes (FLS) are known as the most active cells of rheumatoid arthritis (RA) and are close to the articular cartilage in a position enabling them to invade the cartilage. Macrophage-like synoviocytes and FLS expression of matrix metalloproteinases (MMPs) and their interaction has aroused great interest. The present article studied the expression of CD147, also called extracellular matrix metalloproteinase inducer, on monocytes/macrophages and FLS from RA patients and its potential role in enhancing MMPs and the invasiveness of synoviocytes. Expression of CD147 on FLS derived from RA patients and from osteoarthritis patients, and expression of CD147 on monocytes/macrophages from rheumatic synovial fluid and healthy peripheral blood were analyzed by flow cytometry. The levels of CD147, MMP-2 and MMP-9 mRNA in FLS were detected by RT-PCR. The role of CD147 in MMP production and the cells' invasiveness *in vitro *were studied by the co-culture of FLS with the human THP-1 cell line or monocytes/macrophages, by gel zymography and by invasion assay. The results showed that the expression of CD147 was higher on RA FLS than on osteoarthritis FLS and was higher on monocytes/macrophages from rheumatic synovial fluid than on monocytes/macrophages from healthy peripheral blood. RT-PCR showed that the expressions of CD147, MMP-2 and MMP-9 mRNA was higher in RA FLS than in osteoarthritis FLS. A significantly elevated secretion and activation of MMP-2 and MMP-9 were observed in RA FLS co-cultured with differentiated THP-1 cells or RA synovial monocytes/macrophages, compared with those co-cultured with undifferentiated THP-1 cells or healthy control peripheral blood monocytes. Invasion assays showed an increased number of invading cells in the co-cultured RA FLS with differentiated THP-1 cells or RA synovial monocytes/macrophages. CD147 antagonistic peptide inhibited the MMP production and the invasive potential. Our studies demonstrated that the CD147 overexpression on monocytes/macrophages and FLS in RA patients may be responsible for the enhanced MMP secretion and activation and for the invasiveness of synoviocytes. These findings suggest that CD147 may be one of the important factors in progressive joint destruction of RA and that CD147 may be a potential therapeutic target in RA treatment.

## Introduction

Rheumatoid arthritis (RA) is characterized by chronic proliferative synovitis, with hyperplasia of the synovial lining cells, inflammatory cell infiltration and angiogenesis in the sublining cell layer. Hyperplastic synovial lining cells overproduce such matrix metalloproteinases (MMPs) as MMP-1, MMP-2, MMP-3, MMP-9 and MT1-MMP, which may be involved in tissue remodeling during angiogenesis and cartilage destruction. Articular cartilage at the margins of the articular surface, to which synovium tissue can directly attach, is progressively degraded even in the disease's early stage. As macrophage-like synoviocytes (MLS) and fibroblast-like synoviocytes (FLS) are known as the most active cells and are close to the articular cartilage in a position to invade the cartilage, their expression of MMPs and their interaction have been reported to be related to cartilage destruction. Although IL-1 and tumor necrosis factor alpha are reported by some studies as key regulators of matrix degradation in RA, other molecules, such as CD147, are also thought to have played some regulatory role in RA pathogenesis [1-4]. But very few reports have been presented on CD147 functions in cartilage destruction.

CD147, also called extracellular matrix metalloproteinase inducer and leukocyte activation-associated M6 antigen or HAb18G, was initially identified on the surface of human cancer cells and has been proven to stimulate the adjacent stromal cells to produce several MMPs [5,6]. CD147 is broadly expressed on hemopoietic and nonhemopoietic cell lines [7] and has the function of tissue remodeling. The expression of CD147 has been reported to be upregulated in the synovial membrane of RA patients [8,9]. In our previous study, we found that CD147 was expressed in synovium not only on the fibroblast-like cells, but also on peripheral monocyte-derived CD68^+ ^macrophage-like cells [10].

The study reported here was designed to investigate the expression of CD147 on FLS and THP-1 cells derived from human monocytic leukemia cells [11], from monocytes/macrophages of peripheral blood and from synovial fluid in RA, and to explore the possible functions of CD147 in the pathogenesis of RA.

## Patients and methods

### Patients

Synovium tissues were obtained from ten patients (six with RA, four with osteoarthritis [OA]) at joint replacement surgery or synovectomy, and samples of synovial fluid from knee joints were obtained from ten patients with active RA. All the patients with RA satisfied the 1987 revised diagnostic criteria of the American College of Rheumatology [12]. The mean age of the RA patients was 58 years (range 28–76 years) and the mean disease duration was 10 years. Samples used as the control were obtained from four patients with OA, who met clinical and radiographic American College of Rheumatology criteria for OA. The mean age of these patients was 51 years (range 46–57 years) and the mean disease duration was 12 years. The normal control samples of peripheral blood were taken from ten healthy human donor volunteers, with no significant age or sex differences compared with those of the RA patients. The ethics approval was granted for this study and all the subjects provided their informed consent.

### Cells isolation and culture

Human FLS were isolated by enzymatic dispersion of synovial tissues obtained from RA patients or OA patients. Briefly, tissues were harvested by orthopedic surgeons and collected in sterile PBS. The tissues, with connective tissues and fat removed, were digested with collagenase II (4 mg/ml; Sigma, St Louis, MO, USA) in serum-free DMEM for at least one hour at 37°C. The cell suspension was passed through a nylon mesh and the cells were then collected by centrifugation at 800 × *g *for 5 minutes and were re-suspended in DMEM supplemented with 10% fetal bovine serum (FBS) (Gibco, Grand Island, NY, USA). The harvested cells were cultured in 75 cm^2 ^culture flasks (Costar, Cambridge, MA, USA) with DMEM supplemented with 1% penicillin/streptomycin and 2% L-glutamine (Gibco) and with 10% FBS at 37°C in a humidified atmosphere of 5% CO_2_. When the cells had grown to confluence they were detached with 0.25% trypsin, split in a 1:3 ratio and were re-cultured in DMEM under the same conditions. To eliminate the non-adherent cells, the plated cells were washed thoroughly with PBS. Isolated synoviocytes were cultured in DMEM supplemented with 10% FBS. The cells used for our experiments were at the third to fifth passage, because these cells were more purified than the first and second passages of FLS and had better biological functions than the cells above the fifth passage. The FLS were identified by flow cytometric analysis as a homogeneous population (phenotype: <1% CD14, <1% CD68 and >98% CD90) [13,14].

The human monocyte THP-1 cells (American Type Culture Collection, Manassas, VA, USA) were cultured in RPMI 1640 medium supplemented with 10% FBS, 1% penicillin/streptomycin and 2% L-glutamine at 37°C in a humidified atmosphere of 5% CO_2_. For the induction of cell differentiation, cells (5 × 10^5 ^to 10^6 ^per ml) were seeded in RPMI 1640 serum medium with 200 nM phorbol 12-myristate 13-acetate (PMA) for 24 hours [15]. After incubation, the nonadherent cells were removed by aspiration and the adherent cells were washed with RPMI 1640 three times. THP-1 cells in RPMI 1640 without PMA were used as control (undifferentiated) cells.

Monocytes from heparinized synovial fluid of RA patients were isolated by the Ficoll–Hypaque (Sigma) gradient centrifugation method. The human monocytes were isolated from peripheral blood of healthy humans by Dynal magnetic human CD14 monocyte isolation kits (Dynal Biotech, Oslo, Norway) according to the manufacturer's instructions.

To distinguish the effects of the differentiated and undifferentiated cells, the THP-1 cells were co-cultured with FLS (1 × 10^5 ^cells/35 mm dish) at a cell number ratio of 1.0:1.0 for 24 hours in serum-free DMEM. Undifferentiated THP-1 cells (nonadherent cells) were washed thoroughly with PBS, and FLS (adherent cells) were collected for RT-PCR detection.

### Flow cytometry analysis

Expression of CD147 on the surface of the third to fifth passage of FLS derived from RA or OA synovium tissues were detected by flow cytometry. Cells (5 × 10^5^) were washed three times with PBS and were then treated with fluorescein isothiocyanate-conjugated anti-CD147 monoclonal antibody IgG_1 _or fluorescein isothiocyanate-conjugated Mouse IgG_1 _used as the isotype control (BD Pharmingen, San Diego, CA, USA) for 20 minutes in dark conditions. Cells were washed with PBS and then analyzed by a FACS Calibur flow cytometry, and the data were processed using Cell Quest software (BD Biosciences, San Jose, CA, USA). The positive cell count and the mean fluorescence intensity (MFI) were determined by flow cytometry.

Expression of CD147 on the surface of the differentiated or undifferentiated THP-1 cells and the human monocytes/macrophages from peripheral blood of healthy humans or from synovial fluid of RA patients was also detected by the same method.

### Reverse transcription-polymerase chain reaction

For analysis of MMP-2, MMP-9 and CD147 mRNA levels in FLS derived from RA or OA synovium tissues and for analysis of CD147 mRNA levels in differentiated and undifferentiated THP-1 cells, total RNA was isolated from FLS (four samples with OA FLS, six samples with RA FLS and six samples with RA FLS co-cultured with undifferentiated THP-1 cells) and from THP-1 cells using Trizol (Invitrogen, Carlsbad, CA, USA) according to the manufacturer's instructions. The isolation was followed by the first-strand cDNA synthesis using TrueScript reverse transcriptase (BioRad, Hercules, CA, USA). The cDNA was amplified by PCR using the specific primer set for MMP-2, MMP-9 and CD147 or using glyceraldehyde phosphate dehydrogenase as an internal control.

The sequences of the upstream and downstream primers were respectively as follows: 5'-ATGGGGCTGGAGCACTCCCAAG-3' and 5'-TGTGGCAGCACCAGGGCAGC-3' for MMP-2 (1,070 bp); 5'-GGTCCCCCCACTGCTGGCCCTTCTACGGC-3' and 5'-CACCTCCACTCCTCCCTTTCC-3' for MMP-9 (750 bp); 5'-ACATCAACGAGGGGGAGACG-3' and 5'-GGCTTCAGACAGGCAGGACA-3' for CD147 (492 bp); and 5'-CTGAACGGGAAGCT CACTGG-3' and 5'-TGAGGTCCACCACCCTGTTG-3' for glyceraldehyde phosphate dehydrogenase (313 bp). PCR analysis was performed under the following conditions: denaturation at 94°C for 2 minutes and then 25–30 cycles of denaturation for 30 seconds at 94°C; renaturation for 60 seconds at 56°C for MMP-2, at 58°C for MMP-9 and at 57°C for CD147 and glyceraldehyde phosphate dehydrogenase; and extension for 90 seconds at 72°C. The amplified products were analyzed by agarose gel electrophoresis using 1% gel, followed by ethidium bromide staining.

### Gelatin zymography

Gelatin zymography was performed for detection of MMP activity in conditioned medium. FLS and THP-1 cells were cultured overnight using ordinary serum-containing DMEM. Then FLS, THP-1 cells and the co-culture of FLS and THP-1 cells at the ratio of 1.0:1.0 in serum-free medium were cultured for 24 hours in the presence or absence of HAb18G/CD147 antagonist peptide AP-9 (200 μg/ml), which was produced and characterized in our laboratory based on HAb18G (amino acid sequence YKLPGHHHHYRP) [16-18].

In our previous study, the binding site of AP-9 was confirmed to be the HAB18G/CD147 molecule in the infected 293 cells. AP-9 efficiently blocked the binding sites of HAb18G/CD147 on the cytomembrane and on the unit membrane in the cytoplasm of the 293 cells, and had an inhibitory effect on severe acute respiratory syndrome coronavirus *in vitro *[16]. HAb18G is a hepatoma-associated antigen cloned by hepatoma monoclonal antibody HAb18 from a human hepatocellular carcinoma cDNA library. HAb18G is a highly glycosylated transmembrane protein of 60 kDa with an ectodomain consisting of two regions exhibiting the characteristics of the immunoglobulin superfamily. HAb18G has identical nucleotide acid and amino acid sequences with those of CD147 [17]. HAb18G is a new member of the CD147 family and is abundantly expressed in human hepatoma tissues and on the cell surface of several hepatoma cell lines with a highly invasive potential [5,18-21].

The irrelevant peptide SSP produced in our laboratory, a synthetic epitope peptide of severe acute respiratory syndrome coronavirus S-protein (amino acid sequence FFSTFKCYGVSA) was used as the control. Conditioned media were collected and centrifuged to remove cellular debris and the supernatant was collected and stored at -20°C. Each sample suspension (20 μl) was mixed with SDS sample buffer without reducing agent, followed by detection of the gelatinolytic activity and quantity in the conditioned media by gelatin zymography [22,23]. In brief, the conditioned medium was resolved by SDS-PAGE under non-reducing conditions using 8% separating gel containing 0.1% gelatin. The gels were then washed twice in 2.5% (w/v) Triton X-100 for 30 minutes at room temperature to remove SDS and were incubated in reaction buffer containing 50 mmol/l Tris, 0.2 mol/l NaCl, 5 mmol/l anhydrous CaCl_2 _and 2.5% Triton X-100 for 20 hours at 37°C. The buffer was suitable for proteinase activity, enabling the renatured proteinases to hydrolyze the copolymerized protein substrate in a zone around their position of electrophoresis. The gels were subsequently stained with 0.5% Coomassie blue (R-250) and destained with buffer consisting of 20% methanol, 10% acetic acid and 70% distilled water for 30 minutes to visualize these zones of digestion as light areas against the darkly stained protein background. The zymography gels were scanned and analyzed using US National Institutes of Health Image 1.6 software.

The same method was used to detect the secretion and activation of MMP-2 and MMP-9 of FLS co-cultured with human monocytes/macrophages from peripheral blood of healthy humans or from synovial fluid of RA patients.

Inhibition tests were conducted to verify that the bands of proteolytic activity were MMPs. The MMP inhibitors, ethylenediamine tetraacetic acid (EDTA) (5 mM; Sigma) and 1,10-phenanthroline (20 mM; Sigma), were added separately to the incubation buffer and gels were incubated for 20 hours as described for zymography.

### Invasion assay

The chemotactic cell invasion assay was performed using 24-well transwell units (Costar, Cambridge, NY, USA), each with an 8 μm pore size polycarbonate filter coated with matrigel (5 μg/ml in cold medium) to form a continuous thin layer. Prior to the addition of the cell suspension of FLS, THP-1 cells and co-culture cells (3 × 10^5^) in serum-free medium in the presence or absence of HAb18G/CD147 antagonist peptide AP-9, the dried layer of matrigel matrix was rehydrated with medium without FBS (450 μl). An irrelevant peptide SSP was used in place of HAb18G/CD147 antagonist peptide AP-9 as the control. Using 3T3-conditioned media as the chemoattractant, the cells were then cultured for 24 hours at 37°C in a CO_2 _incubator. The cells remaining in the upper compartment were completely removed with gently swabbing. The filter was fixed and stained with colorimetric H & E. The cells that invaded through the filter into the lower surface of the filter in five microscopic fields of 150 × magnification were counted in each filter. Triplicate samples were conducted and the data were expressed as the average cell number of 15 fields.

The same method was also used to detect the invasive ability of FLS co-cultured with human monocytes/macrophages from peripheral blood of healthy humans or from synovial fluid of RA patients.

### Statistical analysis

All values are expressed as the mean ± standard deviation. Statistical analyses were performed with Student's *t *test using SPSS software, and *P *< 0.05 was considered significant.

## Results

### Expression of CD147 on FLS from RA and OA patients

Expression of CD147 on FLS was evaluated by flow cytometry. Specifically, two parameters of flow cytometry were used: the MFI, which is the quantity of CD147 expression on the surface of each cell, and the percentage of positive staining cells. The result showed that the percentage of positive staining cells of CD147 on RA FLS was higher than that of OA FLS (*P *< 0.05; Table 1). The MFI of CD147 on RA FLS and OA FLS was no different (*P *> 0.05; Table 1).

### Expression of CD147 on THP-1 and monocytes/macrophages

The MFI of CD147 on PMA-induced differentiated human monocyte line THP-1 cells was higher than that on the undifferentiated THP-1 cells (*P *< 0.05; Table 1). The percentage of positive staining cells of CD147 on differentiated and undifferentiated THP-1 cells was no different (*P *> 0.05; Table 1). The MFI of CD147 on the human monocytes/macrophages from synovial fluid of RA patients was higher than that on the human monocytes from peripheral blood of healthy humans (*P *< 0.05; Table 1). The percentage of positive staining cells of CD147 on human monocytes/macrophages from the synovial fluid of RA patients and from peripheral blood of healthy humans was no different (*P *> 0.05; Table 1).

### Expression of CD147, MMP-2 and MMP-9 mRNA in FLS from RA and OA patients

**Figure 1:**
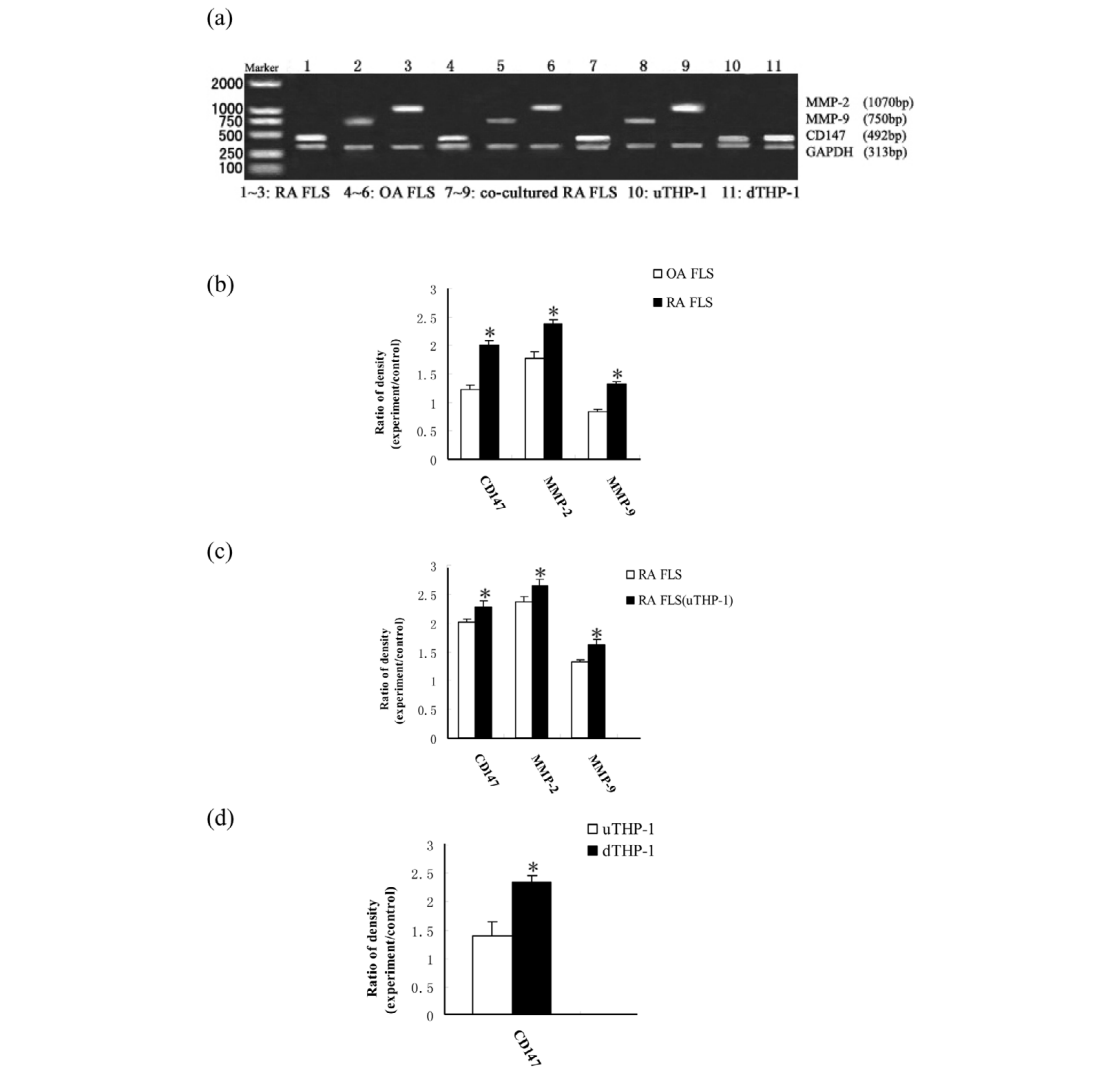
RT-PCR analyses of CD147, MMP-2 and MMP-9 mRNA in fibroblast-like synoviocytes (FLS) from rheumatoid arthritis (RA) patients, from osteoarthritis (OA) patients, and in undifferentiated and differentiated THP-1 cells. Cells were collected and the total RNA isolated by electrophoresis and transferred to a nylon membrane. The membrane was hybridized with CD147-specific, MMP-2-specific and MMP-9-specific cDNAs, and then visualized by autoradiography. **(a) **and **(b) **Expression of CD147, MMP-2 and MMP-9 mRNA in RA FLS (lanes 1, 3 and 2) was higher than that in OA FLS (lanes 4, 6 and 5). (a) and **(c) **Expression of CD147, MMP-2 and MMP-9 mRNA in RA FLS after co-culture with undifferentiated THP-1 cells (lanes 7, 9 and 8) was higher than that in RA FLS (lanes 1, 3 and 2). (a) and **(d) **Expression of CD147 mRNA of differentiated THP-1 cells (dTHP-1, lane 11) was higher than that of undifferentiated THP-1 cells (uTHP-1, lane 10). Glyceraldehyde phosphate dehydrogenase (GAPDH) was used as the inner parameter. Values (ratio of density = experiment/GAPDH) are the means ± standard error (*n *= 4–6). *P < 0.05 (experimental groups versus control groups).

The RT-PCR results were scanned and analyzed using US National Institutes of Health Image 1.6 software. The results indicated that the expressions of CD147, MMP-2 and MMP-9 mRNA were higher in RA FLS than those in OA FLS (Figure 1a,b). The expressions of CD147, MMP-2 and MMP-9 mRNA in RA FLS increased after co-culture with THP-1 cells (Figure 1a,c). The expression of CD147 mRNA in THP-1 cells was enhanced after THP-1 cells were treated with PMA for 24 hours (Figure 1a,d).

### MMP release and activation in co-culture of RA FLS and THP-1 cells

**Figure 2:**
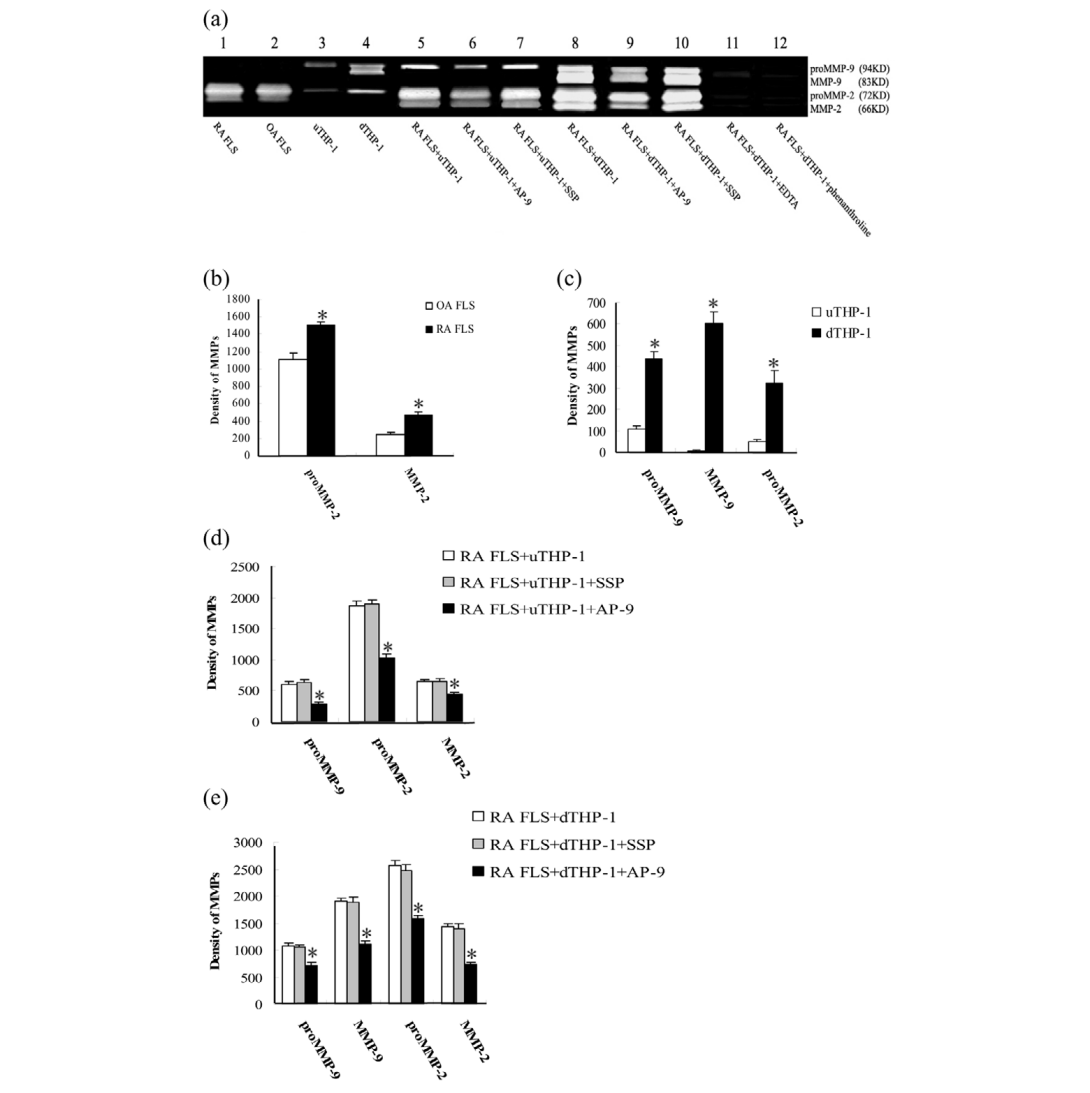
Gelatin zymography of culture medium conditioned by fibroblast-like synoviocytes (FLS) and THP-1 cells. Cells were cultured in serum-free media for 24 hours, and the conditioned media were collected and analyzed for matrix metalloproteinase (MMP) activity by gelatin zymography. **(a) **Secretion and activation of MMPs in FLS from rheumatoid arthritis (RA) and osteoarthritis (OA) patients, THP-1 cells, RA FLS co-cultured with THP-1 cells and in co-cultured cells treated by AP-9 or SSP, and ethylenediamine tetraacetic acid or phenanthroline. Top two bands, MMP-9 gelatinase; lower two bands, MMP-2 gelatinase. **(b) **The value of proMMP-2 and MMP-2 activity of RA FLS was higher than that of OA FLS (six samples with RA FLS, four samples with OA FLS; *P *< 0.05). **(c) **The value of proMMP-9, MMP-9 and proMMP-2 activity of differentiated THP-1 cells (dTHP-1) was higher than that of undifferentiated THP-1 cells (uTHP-1) (*n *= 6, *P *< 0.05). **(d) **The value of proMMP-9, MMP-9 and proMMP-2 activity of RA FLS co-cultured with uTHP-1 was higher than that of co-cultured cells treated by AP-9 (*n *= 6, *P *< 0.05), while no inhibitory effects were found in irrelevant peptide SSP (*n *= 6, *P *> 0.05). **(e) **The value of proMMP-9, MMP-9, proMMP-2 and MMP-2 activity of RA FLS co-cultured with dTHP-1 was higher than that of co-cultured cells treated by AP-9 (*n *= 6, *P *< 0.05), while no inhibitory effects were found in irrelevant peptide SSP (*n *= 6, *P *> 0.05). Values are the means ± standard error (*n *= 4–6). *P < 0.05 (experimental groups versus control groups).

Gelatin zymography showed that the secretion and activation of MMP-2 and MMP-9 increased in the co-culture of RA FLS and differentiated THP-1 cells compared with those in the co-culture of RA FLS and undifferentiated THP-1 cells (*P *< 0.05; Figure 2). The secretion and activation of proMMP-2 and MMP-2 of RA FLS were higher than those of OA FLS (*P *< 0.05; Figure 2a,b). An increase was observed in the release of MMP-9 in the differentiated THP-1 cells compared with that in the undifferentiated THP-1 cells (*P *< 0.05; Figure 2a,c).

In inhibition tests, gelatin zymography showed that the proteinases were all inhibited by EDTA and phenanthroline (Figure 2a), which verified that these enzymes were all MMPs.

### MMPs release and activation in co-culture of RA FLS and human monocytes/macrophages

**Figure 3:**
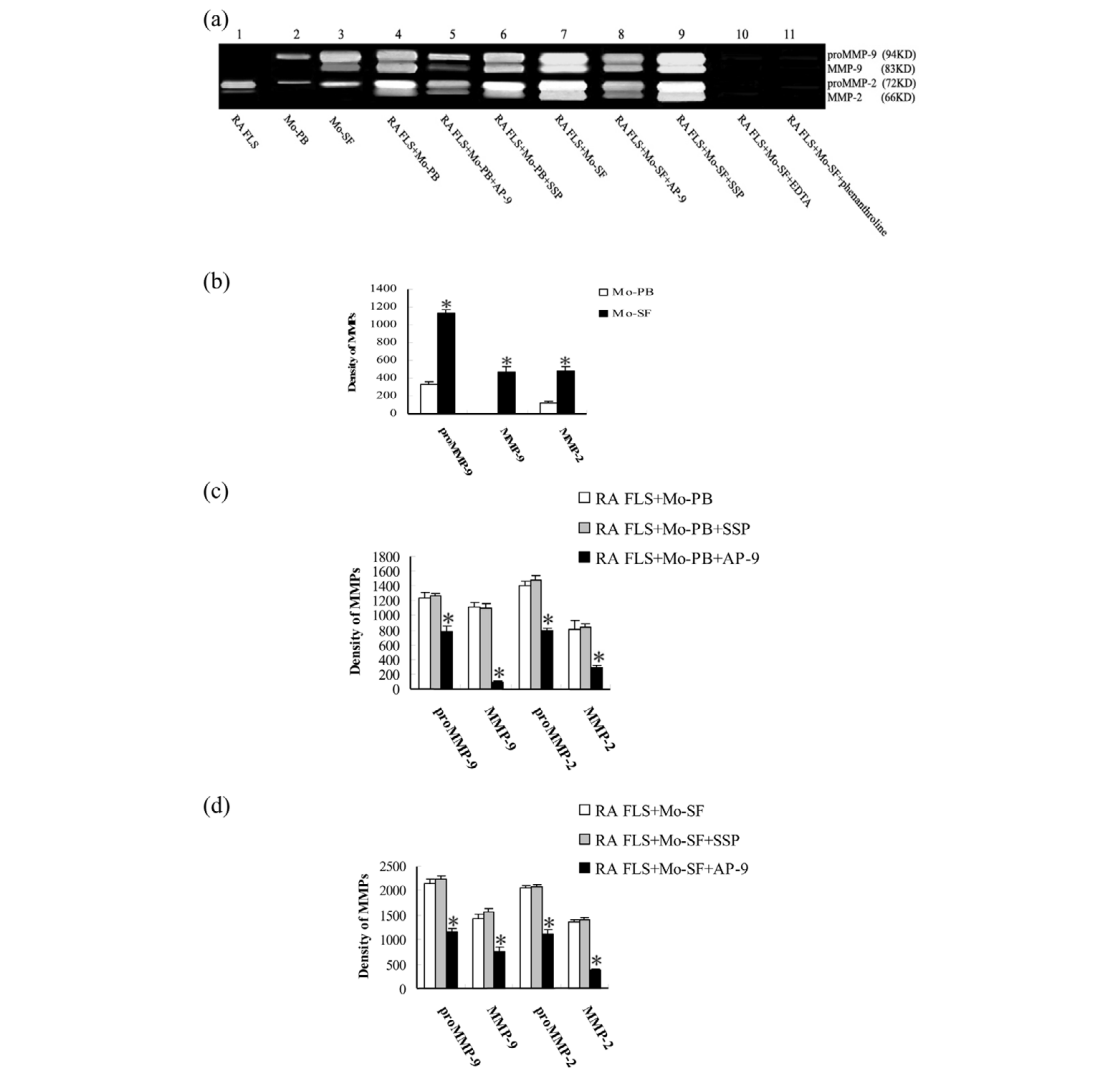
Gelatin zymography of culture medium conditioned by fibroblast-like synoviocytes (FLS) and monocytes/macrophages. Cells were cultured in serum-free media for 24 hours, and the conditioned media were collected and analyzed for matrix metalloproteinase (MMP) activity by gelatin zymography. **(a) **Secretion and activation of MMPs in rheumatoid arthritis (RA) FLS, monocytes/macrophages, RA FLS co-cultured with monocytes/macrophages, and in co-cultured cells treated by AP-9 or SSP, and ethylenediamine tetraacetic acid or phenanthroline. Top two bands, MMP-9 gelatinase; lower two bands, MMP-2 gelatinase. **(b) **The value of proMMP-2 and MMP-2 activity of monocytes from peripheral blood of healthy humans (Mo-PB) was lower than monocytes/macrophages from synovial fluid of RA patients (Mo-SF) (*n *= 10, *P *< 0.05). **(c) **The value of proMMP-9, MMP-9, proMMP-2 and MMP-2 activity of RA FLS co-cultured with Mo-PB was higher than that of co-cultured cells treated by AP-9 (*n *= 6, *P *< 0.05), while no inhibitory effects were found in irrelevant peptide SSP (*n *= 6, *P *> 0.05). **(d) **The value of proMMP-9, MMP-9, proMMP-2 and MMP-2 activity of RA FLS co-cultured with Mo-SF was higher than that of co-cultured cells treated by AP-9 (*n *= 6, *P *< 0.05), while no inhibitory effects were found in irrelevant peptide SSP (*n *= 6, *P *> 0.05). Values are the means ± standard error (*n *= 6–10). *P < 0.05 (experimental groups versus control groups).

The gelatin zymography results showed that MMP-9 and MMP-2 secretion and activation increased in the co-culture of RA FLS and human monocytes/macrophages from synovial fluid of RA patients compared with those in the co-culture of RA FLS and human monocytes from normal peripheral blood (*P *< 0.05; Figure 3). The secretion and activation of proMMP-9, MMP-9 and MMP-2 of human monocytes/macrophages from synovial fluid of RA patients were higher than those of human monocytes from normal peripheral blood (*P *< 0.05; Figure 3a,b).

The inhibition tests showed the same results as those in gelatin zymography of RA FLS and THP-1 cells (Figure 3a).

### Invasive potential of cells in co-culture of RA FLS and human monocytes/macrophages or THP-1 cells

**Figure 4:**
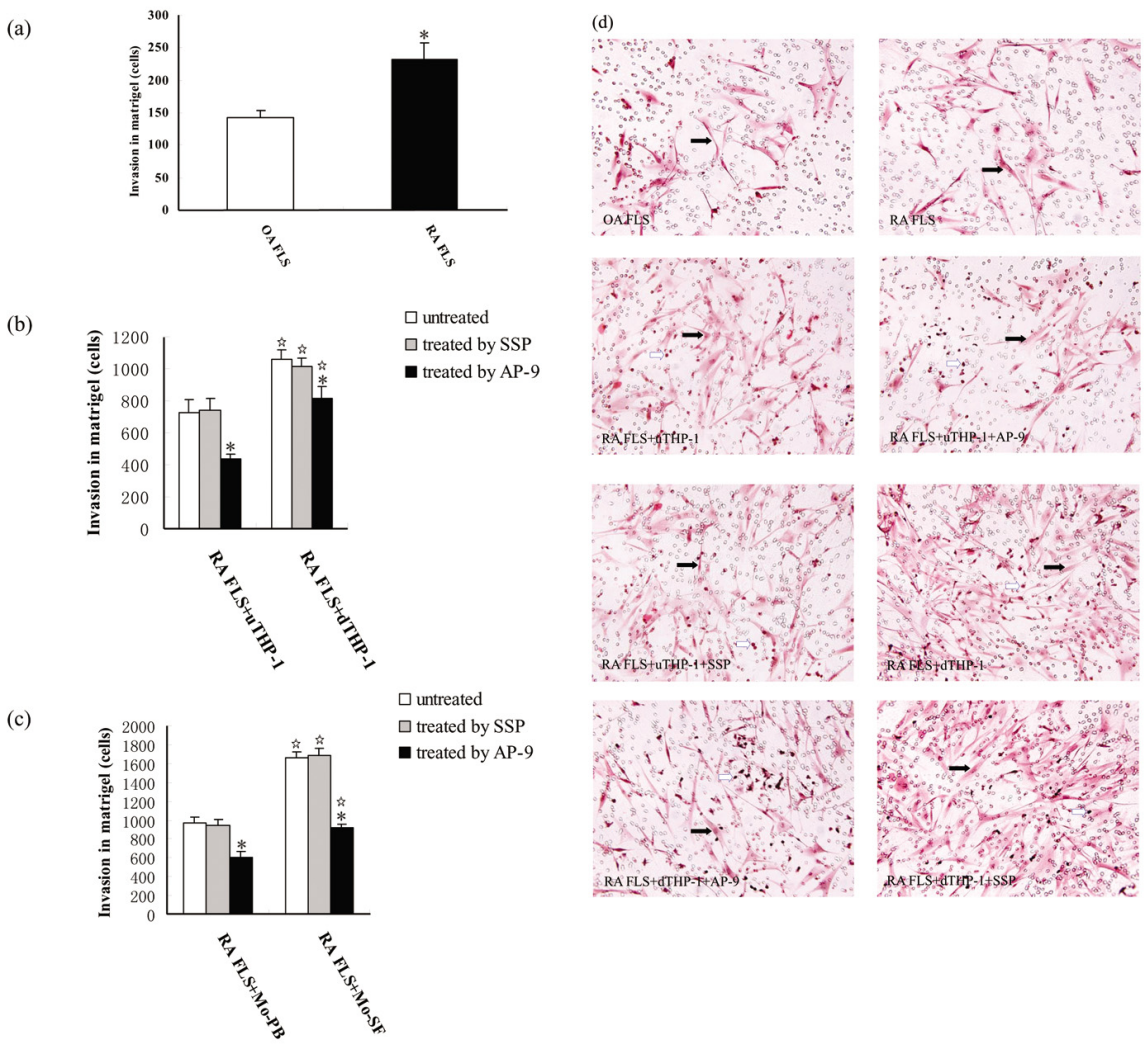
Invasive potential in co-culture of rheumatoid arthritis (RA) fibroblast-like synoviocytes (FLS) and monocytes/macrophages or THP-1 cells, and RA/osteoarthritis (OA) FLS alone. The invasive potential was evaluated in transwell chambers as described in Patients and methods. Briefly, cells were suspended in serum-free media supplemented with or without AP-9 or SSP (200 μg/ml) and seeded into the upper side of the matrigel (5 μg/ml)-coated chambers. After incubation for 24 hours at 37°C, the number of invaded cells was determined using a colorimetric H & E assay. Black arrow, FLS; white arrow, THP-1 cells (f × 200). **(a) **and **(d) **The invasive potential of RA FLS was higher than that of OA FLS (*P *< 0.05; six samples with RA FLS, four samples with OA FLS). **(b) **and **(d) **the invasive potential of RA FLS co-cultured with undifferentiated THP-1 cells (uTHP-1) and differentiated THP-1 cells (dTHP-1) and the invasive potential of co-cultured cells inhibited by AP-9 (*P *< 0.05, RA FLS+ uTHP-1 vs RA FLS+ uTHP-1+ AP-9 and RA FLS+ dTHP-1 vs RA FLS+ dTHP-1+ AP-9; *n *= 6, respectively), while no inhibitory effects were found in irrelevant peptide SSP (*P *> 0.05, RA FLS+ uTHP-1 vs RA FLS+ uTHP-1+ SSP and RA FLS+ dTHP-1 vs RA FLS+ dTHP-1+ SSP; *n *= 6, respectively). **(c) **The invasive potential of RA FLS co-cultured with monocytes/macrophages from synovial fluid of RA patients (Mo-SF) and monocytes from peripheral blood of healthy humans (Mo-PB) and the invasive potential of co-cultured cells inhibited by AP-9 (*n *= 10, *P *< 0.05), while no inhibitory effects were found in irrelevant peptide SSP (*n *= 10, *P *> 0.05). Values are the means ± standard error (*n *= 4–10). *indicates *P* < 0.05 (experimental groups versus control groups). Stars indicate *P *< 0.05 (RA FLS co-cultured with dTHP-1 versus RA FLS co-cultured with uTHP-1, and RA FLS co-cultured with Mo-SF versus RA FLS co-cultured with Mo-PB).

A higher number of RA FLS (232 ± 26.29 cells/filter) were found to have invaded through transwell chambers than that of OA FLS (142.3 ± 11.02 cells/filter) (*P *< 0.05; Figure 4a,d). A higher number of RA FLS co-cultured with differentiated THP-1 cells (1055.67 ± 63.06 cells/filter) were found to have invaded through transwell chambers than that of RA FLS co-cultured with undifferentiated THP-1 cells (726 ± 83.86 cells/filter) (*P *< 0.05; Figure 4b,d). RA FLS co-cultured with human monocytes/macrophages from synovial fluid of RA patients were found to have a higher number invade through the transwell chambers (1655.67 ± 63.06 cells/filter) than that of RA FLS co-cultured with human monocytes from normal peripheral blood (972.67 ± 59.21 cells/filter) (*P *< 0.05; Figure 4c).

### Effect of HAb18G/CD147 antagonistic peptide AP-9 on MMP release and activation and on the invasion processes

HAb18G/CD147 antagonistic peptide AP-9 (200 μg/ml) was found to have inhibitory effects on the MMP release and activation of co-cultured RA FLS and THP-1 cells (differentiated and undifferentiated), and on co-cultured RA FLS and monocytes/macrophages from healthy peripheral blood or rheumatic synovial fluid (*P *< 0.05; Figures 2a,d,e and 3a,c,d). However, the irrelevant peptide SSP was not found to have inhibitory effects (*P *> 0.05; Figures 2a,d,e and 3a,c,d).

The invasion block assay showed that the amounts of cells invaded through the matrigel-coated filter decreased after the treatment with AP-9 (200 μg/ml) for 24 hours in RA FLS co-cultured with THP-1 cells (undifferentiated and differentiated). The inhibitory rate of invasive potential in RA FLS co-cultured with undifferentiated THP-1 cells and RA FLS co-cultured with differentiated THP-1 cells was 39.53% and 22.8%, respectively (*P *< 0.05; Figure 4b,d), while no inhibitory effects were found in the irrelevant peptide SSP (*P *> 0.05; Figure 4b,d). The inhibitory rate of invasive potential in RA FLS co-cultured with human monocytes/macrophages from the synovial fluid of RA patients and RA FLS co-cultured with human monocytes from normal peripheral blood was 44.74% and 37.73%, respectively (*P *< 0.05; Figure 4c), while no inhibitory effects were found in the irrelevant peptide SSP (*P *> 0.05; Figure 4c).

## Discussion

FLS and MLS at the cartilage-pannus junction of RA patients contribute most to the joint destruction and they produce a great amount of MMPs. These enzymes act in a synergistic manner to destroy connective tissue components [24,25]. The combination of MMPs is capable of degrading all essential extracellular matrix components of the synovial membrane, articular cartilage and subchondral bone. Imbalanced activity between MMPs and tissue inhibitors of metalloproteinases caused by enhanced expression of CD147 eventually leads to joint destruction in RA [26-28].

CD147 is more highly expressed on human carcinoma cells than on normal cells and its expression correlates with the MMP expression level and the cancer invasive potential [29-32]. The aggressive characteristics of RA synovium are similar to those of neoplastic tissues [33]. The proliferating mass of synoviocytes locally invades the cartilage and bone, destroying the joint. MMPs, mainly produced in FLS, play a central role in arthritic joint destruction. Beyond the proof that the expressions of MMP-2 and MMP-9 as well as the invasive potential of RA FLS are higher than those of OA FLS, we have also found in this study, by cell isolation and culture, that the expression of CD147 on RA FLS is higher than that on OA FLS.

The monocyte/macrophage lineage, especially MLS, is also known to play an important role in RA pathogenesis. In this study, we found that expressions of CD147, MMP-2 and MMP-9 of differentiated THP-1 cells or monocytes/macrophages from synovial fluid of RA patients are higher than those of undifferentiated THP-1 cells or monocytes from peripheral blood of healthy humans. PMA might be a main upregulative factor that enhances expression of CD147 on the differentiated THP-1 cells. Some cytokines in the synovial fluid of RA patients, such as interferon gamma and granulocyte–macrophage colony-stimulating factor, may also enhance the expression of CD147 on the monocytes/macrophages from synovial fluid of RA patients. The overexpression of CD147 on differentiated monocytes/macrophages suggests that CD147 may be important in the early phases of direct cell migration and of both autocrine and paracrine stimulation of MMP expression. However, further work is needed to test this supposition.

In our previous study, we found that CD147 was expressed predominantly on the MLS and FLS in the lining and sublining layers of RA synovium, and macrophages acted as the amplifier of the pathogenetic cascade in RA via the increase of MMP production by interacting macrophages with fibroblasts [10]. To explore the effect of cell–cell interaction on the production of MMPs and the invasiveness of RA synoviocytes, RA FLS were co-cultured with THP-1 cells in the present study. We have found that the expression of CD147 on RA FLS is enhanced by cell–cell interaction, and that the secretion and activation of MMPs and the invasive potential of RA FLS are also enhanced. These results suggest that the overexpression of CD147 on macrophages may accelerate the production of MMPs and the invasive ability of RA FLS by cell–cell interaction. The overexpression of CD147 on FLS and the monocyte/macrophage lineage, especially on MLS, suggests that CD147 may be important in both autocrine and paracrine stimulation of MMPs. From the findings that homophilic CD147 binding has occurred in the context of both heterotypic and homotypic cell–cell interactions and that CD147 can be a receptor in itself to induce MMP production, not only in primary fibroblast cells but also in tumor cells themselves [34], we presume that the increased expression of CD147 on FLS and the monocyte/macrophage lineage, especially on MLS, in RA synovium could possibly induce MMP production through interaction.

To further confirm the effect of cell–cell interactions in rheumatic joints, monocytes/macrophages from synovial fluid of RA patients and from peripheral blood of healthy humans were co-cultured with RA FLS in this study. The environments of co-cultured RA FLS and monocytes/macrophages from synovial fluid of RA patients or from peripheral blood of healthy humans are more like the real environment *in vivo *than that of co-cultured RA FLS and THP-1 cells. The results from this co-culture were consistent with those of RA FLS co-cultured with THP-1 cells, indicating that the overexpression of CD147 induces elevated levels of MMPs and their activated forms in RA FLS – and the elevated levels of MMPs in turn enhance the invasive ability of RA FLS.

In this study, CD147 antagonistic peptide was added in the condition medium of co-cultured cells. We have found that the addition of CD147 antagonistic peptide has some inhibitory effect not only on the MMP production, but also on the invasive potential of the co-cultured FLS. The inhibition of MMP-2 and MMP-9 production by CD147 antagonistic peptide indicates that CD147 may induce MMP-2 and MMP-9 production. The mechanism by which CD147 regulates MMP-2 and MMP-9 production is largely unclear, although a recent study has suggested that the mitogen-activated protein kinase p38 pathway may be involved during MMP induction in dermal fibroblasts [35]. Our previous studies have indicated that overexpression of HAb18G/CD147 enhances metastatic potentials in human hepatoma cells by disrupting the regulation of store-operated Ca^2+ ^entry by nitric oxide/cGMP. CD147 is required to mediate the effect of HAb18G/CD147 on the secretion and activation of MMPs and metastasis-related processes in human hepatoma cells by disrupting the regulation of nitric oxide/cGMP-sensitive intracellular Ca^2+ ^mobilization [19,20]. These findings also indicate that CD147 is involved in promoting the secretion and activation of MMPs, which in turn increases the invasive potential of FLS. These results also suggest that CD147 expression may be correlated to MMP (MMP-2, MMP-9) secretion, activation and invasive potential in RA FLS.

Very low levels of CD147 have been reported in most normal adult tissues, including the epidermis, retinal pigment epithelium, and breast lobules and ductules. CD147 may therefore play a physiologic role in tissue remodeling by inducing MMPs [36-39]. A correlation between CD147 and progressive joint destruction in RA is suggested in this study based on the findings that the overexpression of CD147 on monocytes can facilitate enhancement of the production of MMPs and the invasion ability of FLS, and that CD147 antagonistic peptide can block the enhancement.

## Conclusion

We conclude in this study that the increased expression of CD147 on FLS and macrophage-like cells in RA may be responsible for the elevated MMP secretion and the activation and the invasive potential of the cell, all of which may contribute to the cartilage and bone destruction characteristic of RA. These findings, together with a better understanding of the relationship between CD147 and RA, and of the possible mechanism and regulation of the effect of CD147 on MMP production, will help in the development of innovative therapeutic interventions for RA.

## Abbreviations

bp = base pairs; DMEM = Dulbecco's modified Eagle's medium; EDTA = ethylenediamine tetraacetic acid; FBS = fetal bovine serum; FLS = fibroblast-like synoviocytes; H & E = hematoxylin and eosin; IL = interleukin; MFI = mean fluorescence intensity; MLS = macrophage-like synoviocytes; MMP = matrix metalloproteinase; OA = osteoarthritis; PBS = phosphate-buffered saline; PCR = polymerase chain reaction; PMA = phorbol 12-myristate 13-acetate; RA = rheumatoid arthritis; RT = reverse transcriptase.

## Competing interests

The authors declare that they have no competing interests.

## Authors' contributions

PZ participated in the design of the study and drafted the manuscript. NL participated in the design of the study, performed the invasion and gel zymography assays, and drafted the manuscript, and he is one of the co-first authors. ZS carried out the flow cytometry assay and helped to draft the manuscript, and is one of the co-first authors. JZ performed the invasion and gel zymography assays. ZW performed the statistical analysis. YY performed the RT-PCR. JD carried out the flow cytometry assays. ZC participated in the design of the study and helped draft the manuscript. All authors read and approved the final manuscript.

## Figures and Tables

**Table 1:** Results of expressions of CD147 on fibroblast-like synoviocytes (FLS) and monocytes/macrophages

	Osteoarthritis FLS	RA FLS	Undifferentiated THP-1 cells	PMA-induced differentiated THP-1 cells	Monocytes/macrophages from peripheral blood of healthy humans	Monocytes/macrophages from synovial fluid of RA patients
Percentage of positive staining cells (%)	88.75 ± 2.32	97.17 ± 0.6*	99.23 ± 0.47	99.33 ± 0.38	99.18 ± 0.55	99.27 ± 0.39
Mean fluorescence intensity	192.72 ± 12.67	193.33 ± 15.83	202.1 ± 19.25	286.61 ± 31.63^#^	78.32 ± 18.25	136.37 ± 28.16^‡^
Number	4	6	6	6	10	10

Values presented as the mean ± standard error (*n *= 4–10 independent samples per group). Expressions of CD147 on FLS from osteoarthritis or rheumatoid arthritis (RA) patients and monocytes/macrophages (including undifferentiated THP-1/phorbol 12-myristate 13-acetate [PMA]-induced differentiated dTHP-1 cells, monocytes/macrophages from synovial fluid of rheumatoid arthritis patients or from peripheral blood of healthy humans) were measured by flow cytometry using fluorescein isothiocyanate-labeled anti-CD147 mAb. Analysis was conducted on a FACS™ brand flow cytometer. Two parameters of FLS and THP-1 cells were used: the percentage of positive staining cells, and the mean fluorescence intensity (denoting the quantity of CD147 expression on the surface of each cell).

*P < 0.05 versus OA FLS; ^#^*P *< 0.05 versus undifferentiated THP-1 cells; ^‡^*P *< 0.05 versus monocytes from peripheral blood of healthy humans.
